# Label free proteomics and systematic analysis of secretome reveals effector candidates regulated by SGE1 and FTF1 in the plant pathogen *Fusarium oxysporum* f. sp. *cubense* tropical race 4

**DOI:** 10.1186/s12864-020-6695-9

**Published:** 2020-04-03

**Authors:** Shixue Zhao, Bang An, Yanhua Guo, Xingrong Hou, Hongli Luo, Chaozu He, Qiannan Wang

**Affiliations:** 1 Hainan Key Laboratory for Sustainable Utilization of Tropical Bioresource, College of Tropical Crops, Hainan University, Haikou, Hainan 570228 People’s Republic of China; 2 College of Fisheries and Life Science, Hainan Tropical Ocean University, Sanya, Hainan 572022 People’s Republic of China

**Keywords:** *F. oxysporum f. sp. cubense*, Secretome, Label free proteomics, Effectors

## Abstract

**Background:**

Phytopathogens secreted effectors during host colonization to suppress or trigger plant immunity. Identification of new effectors is one of the research focuses in recent years. There is only a limited knowledge about effectors of *Fusarium oxysporum* f. sp. *Cubense* tropical race 4 (Foc TR4), the causal agent of wilt disease in Cavendish banana.

**Results:**

Two transcription factors, SGE1 and FTF1, were constitutively over-expressed in Foc TR4 to partially mimic the in-planta state. Secreted proteins with high purity were prepared through a two-round extraction method. Then the secretome were analyzed via label free proteomics method. A total of 919 non-redundant proteins were detected, of which 74 proteins were predicted to be effector candidates. Among these candidates, 29 were up-regulated and 13 down-regulated in the strain over-expressing SGE1 and FTF1, 8 were up-regulated and 4 down-regulated in either SGE1 or FTF1 over expression strain.

**Conclusions:**

Through label free proteomics analysis, a series of effector candidates were identified in secretome of Foc TR4. Our work put a foundation for functional research of these effectors.

## Background

Fungal disease is one of the major threats to global food security. In the long periods of co-evolution with plant hosts, pathogenic fungi have evolved complex mechanisms to cope with plant immune systems. One of the strategies is to secret effectors. Effectors are defined as proteins that are secreted by bacteria, oomycetes, and fungi to facilitate infection and/or trigger defense responses in host plant [1]. Bacteria employ specialized secretion systems, such as the type III secretion system, to directly inject effectors into host cell cytoplasm; and signals sequence are widely existed in bacterial effectors [2]. In oomycete pathogens, there are also consensus N-terminal sequence motifs in effectors, such as RXLR, LFLAK, and CHXC amino acid sequences. Besides, oomycete pathogens secret effectors via the differentiated cells named as haustoria [3]. In fungal pathogens, no consensus sequence motifs were identified in diverse effectors; furthermore, fungal pathogens secret effectors via multiple systems including appressorium, invasive hyphae or haustoria [3]. These facts contribute to the diversity of fungal effectors and make it difficult to predict potential effectors.

*Fusarium oxysporum* spp. are world wide spread soil-borne pathogens and have a remarkably broad host range. In *F. oxysporum*, effectors are required for full virulence of the pathogens to their hosts. Via analyzing the xylem sap proteome of the infected tomato plantlets, a group of cysteine-rich effectors named as SIX (secreted in xylem) were firstly identified in *F. oxysporum* f. sp. *lycopersici* (Fol) [4]. These SIX proteins display little homology with other known proteins. Fungal effectors were divided into apoplast and cytoplasm effectors, which function in the extracellular matrix and inside the host cells, respectively [1]; hence, investigation of plant xylem sap proteome alone might lead to the ignorance of the effectors that was taken up by plant cells. Meanwhile, effectors with extremely low abundance in xylem sap might also be neglected due to the detection range limit of mass spectrograph. In vitro culture and appropriate induction could enhance enrichment of secretome; however, most of effector genes are induced or specifically expressed during in-planta status [5, 6]. Thus, successful mimic of the *in planta* status is important for the induction of the expression of effectors during in vitro culture, and make it possible for identification of potential effectors from secretome.

Previous works showed that some transcription factors play key roles in regulating the transcription of effector encoding genes. In *Ustilago maydis*, several types of transcription factors, including the heterodimer bE/bW and the forkhead transcription factor Fox1, regulate the expression of effector genes [7, 8]. In *Leptosphaeria maculans* and *Stagnospora nodorum*, homologs of StuA are involved in regulation of several effector genes [9, 10]. The transcription factor SGE1 (SIX gene expression 1) was found to regulate the expression of SIX effectors of Fol in vivo [11]. In other *F. oxysporum* species, SGE1 is also required for the expression of *SIX* genes and secondary metabolite genes [12, 13]. SGE1 is the ortholog of the conserved fungal transcription factor Wor1 from *Candida albicans* and *Histoplasma capsulatum*, which regulate the morphological transition and is associated with virulence towards humans [14, 15]. In Fol, genomic researches revealed that effector genes reside on an accessory chromosome, named as pathogenic chromosome, which can be transferred horizontally between strains [16]. In addition to *SGE1* which resides on the core genome, a group of transcription factors coding genes named as *FTF* (Fusarium transcription factor) are found to reside on both core and the pathogenic chromosomes of Fol [17]. In *F. oxysporum* f. sp. *Phaseoli*, *FTF1* is up-regulated during infection to runner bean plants and is required for pathogenicity of the pathogen [18]. Knocking down or knocking out of the FTF coding genes suggested that FTF regulate pathogenicity mainly by controlling the expression of effectors [19]. Expression profile analysis showed that the transcription levels of *SGE1* and *FTF1* both increase during infection processes; and constitutive expression of FTF1, FTF2 or SGE1 induced expression of a large overlap set of known effector genes in Fol, suggesting an interaction of these transcription factors [17]. But whether there are potential effectors regulated by SGE1 or FTF in Foc TR4 is still elusive.

*F. oxysporum* f. sp. *cubense* (Foc) is the agent of banana (*Musa* spp.) wilt disease (also named as ‘panama disease’). Among the races of Foc, Foc tropical race 4 (Foc TR4) is a worldwide spread pathogen causing disaster to *Cavendish* banana plantation [20]. Label-free quantitative proteomics is a powerful technique with higher proteome coverage capacity and dynamic range in comparison with other proteomic technologies [21]. In the present study, to explore new effector candidates of Foc TR4, the SGE1 and FTF1 over-expression strains were constructed respectively; then the secretome of the strains were analyzed via label-free quantitative proteomics technique and the effector candidates were predicted via systematic analysis. This work provides a foundation for investigation of function of these newly identified effectors.

## Results

### Generation of the SGE1 and FTF1 over-expression strains

**Fig. 1:**
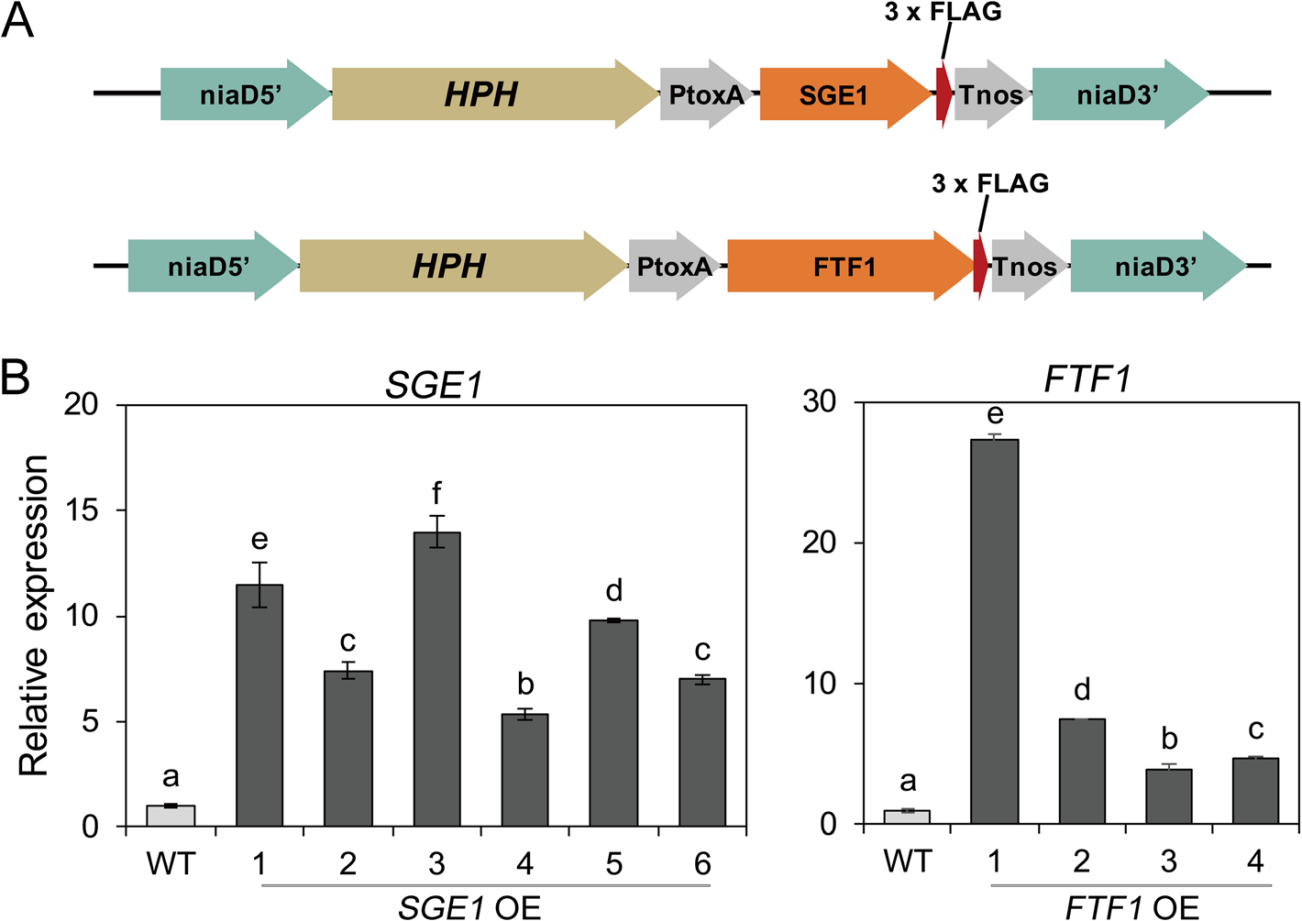
Generation of the SGE1 and FTF1 over-expression transformants. **a** The diagram of over-expression vectors. The locus of nitrate reductase (*niaD*) was used as the targeted integration of reporter gene constructs. **b** Quantitative RT-PCR analysis of relative gene transcription levels in Foc TR4 strains. WT: wild type; OE: over-expression transformants

For generation of the SGE1 and FTF1 over expression (OE) transformants, the ORFs of the genes were ligated into the downstream of the strong promoter *ToxA* of the plasmid (Fig. 1a); and hygromycin phosphotransferase conferring resistance to Hygromycin B was used as the selection marker. After protoplast transformation, the transformants resistant to 300 mg mL^− 1^ Hygromycin B were selected for the diagnostic PCR analysis. A total of 6 transformants were identified for successful integration of the *SGE1* expressing cassette into the genome, and 4 transformants for the *FTF1* (data not shown). After culture on potato dextrose agar (PDA) medium for 3 days, the mycelium of the transformants were collected for RNA extraction and cDNA synthesis. The relative expression levels of *SGE1* and *FTF1* were estimated with qRT-PCR. The results showed that transcription levels of *SGE1* and *FTF1* were significantly increased for at least 5 folds in the corresponding OE transformants (Fig. 1b). Then the transformants were named as SGE1 OE and FTF1 OE respectively, and the two transformants with the highest expression levels (SGE1 OE3 and FTF1 OE1) were selected for further research. A wild type (WT) was used as a reference sample for the following analysis.

### Secretome with high purity were obtained

**Fig. 2:**
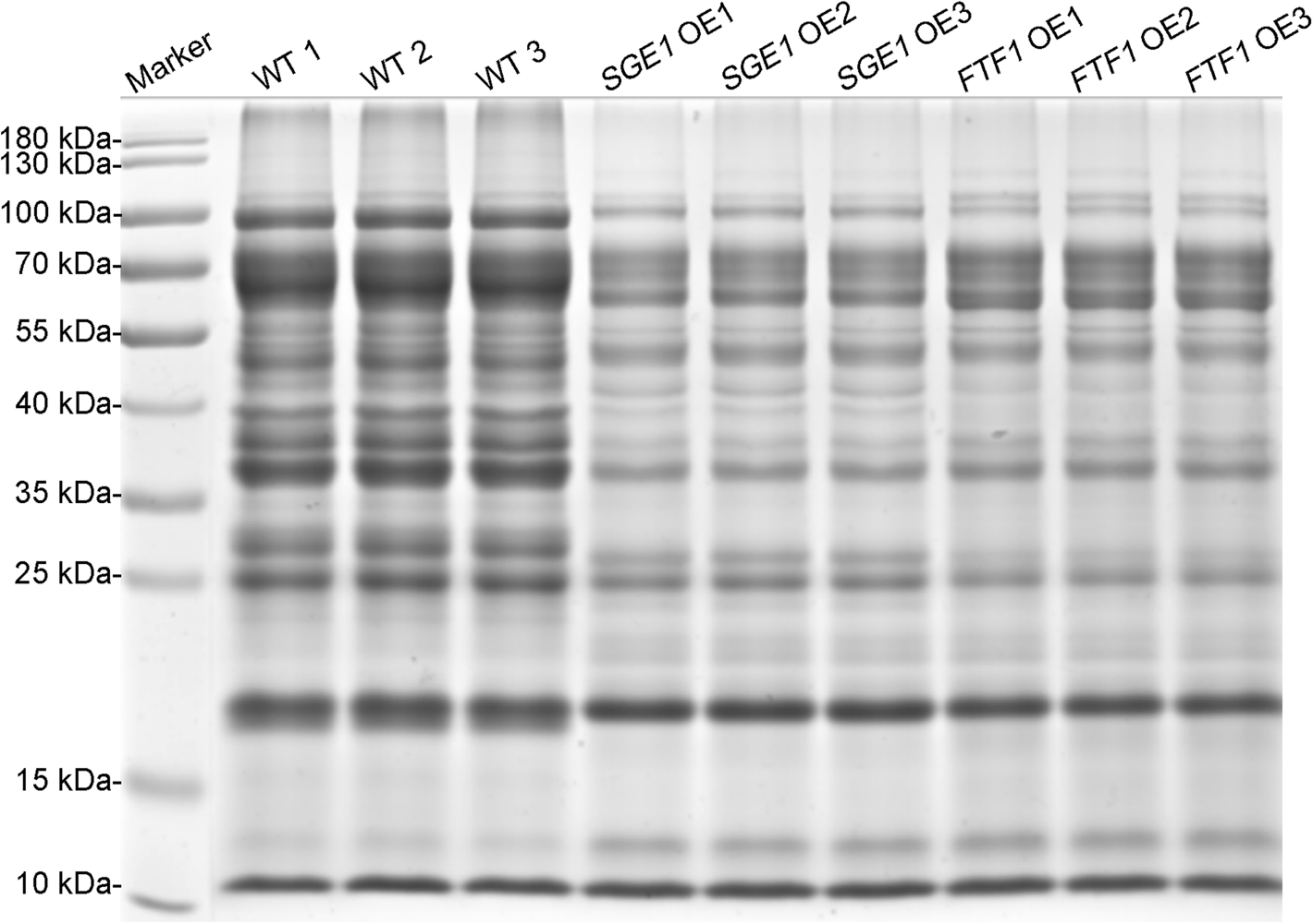
SDS-PAGE analysis of extracellular proteins of Foc TR4 strains. WT: wild type; OE: over-expression transformants

To obtain sufficient secreted proteins with high purity, the two-round extraction and purification method were employed in the present study. 20 μg of purified protein of each sample was examined in 12% SDS-PAGE. The results showed that the purified protein samples were with high quality and with little impurities (Fig. 2).

### Label-free quantitative proteomics analysis and prediction of effectors

**Fig. 3:**
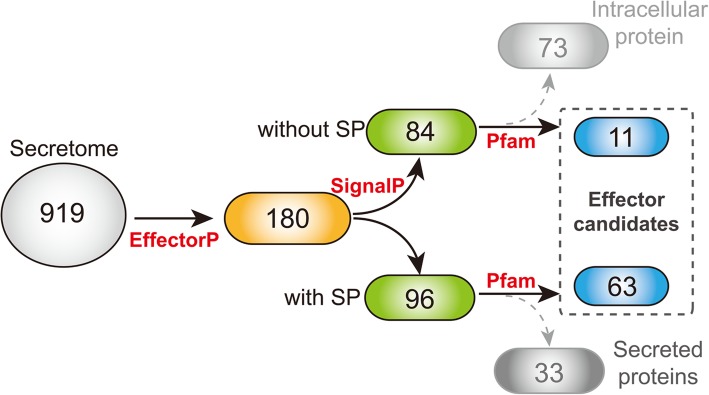
Effector prediction from secretome and analysis pipeline. SP: signal peptides

Label-free quantitative proteomics was used to compare secretome from the three groups of samples: WT, SGE1 OE and FTF1 OE. In total, 919 non-redundant proteins were detected based on the identification of one or more unique peptides (Table S1). The probable effectors were predicted based on the following procedures (Fig. 3). Firstly, 180 of the 919 proteins were identified with EffectorP 2.0 as primary candidates. Secondly, the 180 candidates were divided into two subgroups based on the existence of signal peptides: 96 candidates with SP and 84 without SP. Thirdly, the two subgroups of candidates were searched for known functional domains using Pfam database respectively. According to the results, 33 proteins with signal peptides were predicted to be apoplastic enzymes, and 73 proteins without SP were predicted to be intracellular functional proteins; then these 106 proteins were excluded from the candidates. Finally, a total of 74 candidates were predicted to be effectors.

**Fig. 4:**
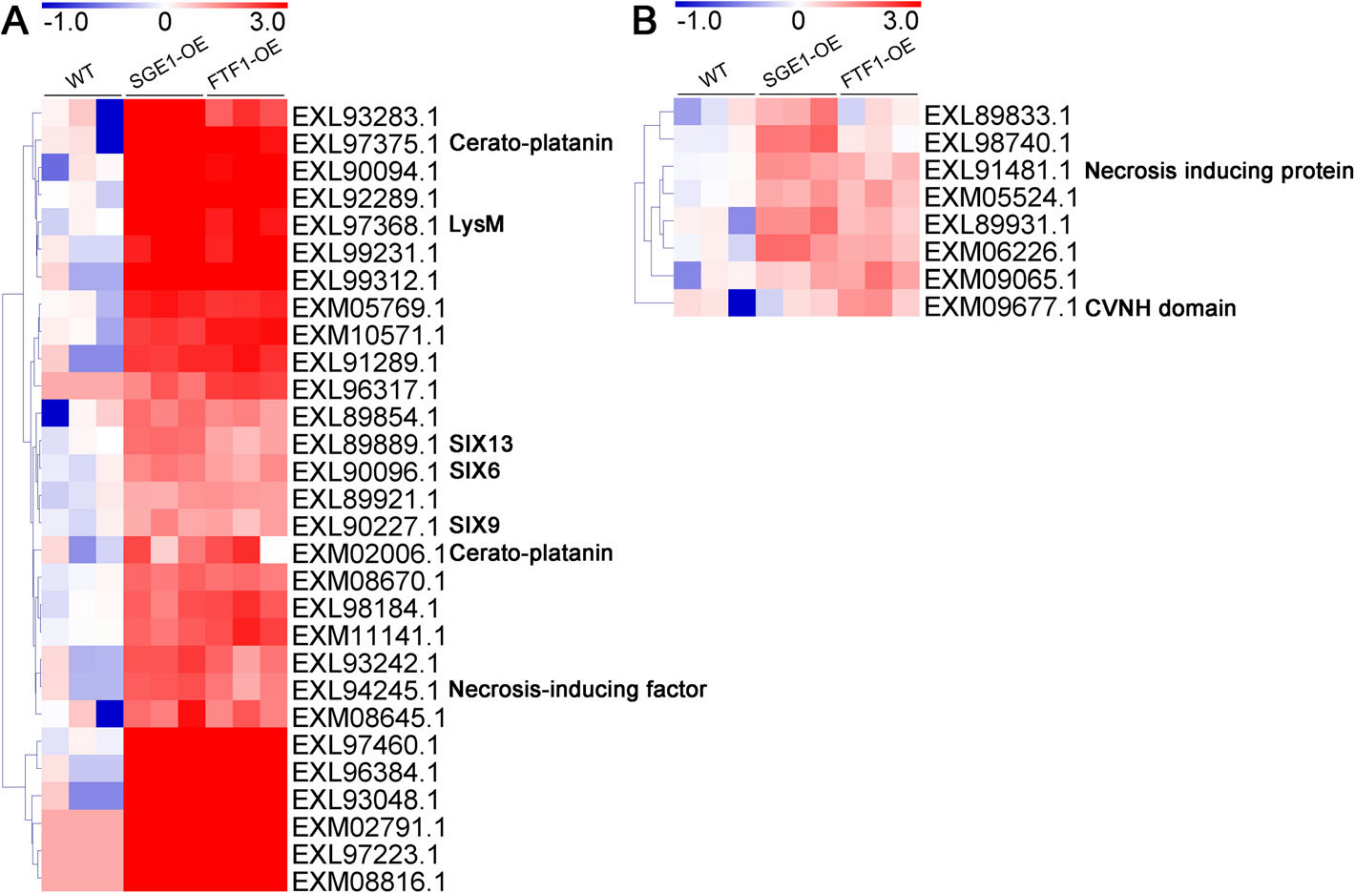
Profiles of the up-regulated effector candidates. Fold changes of protein abundance were calculated using the mean value of wild type samples as reference. The heatmaps were created based on the Log_2_(Fold change) values. **a** Proteins up-regulated in both over-expression samples. **b** Proteins up-regulated in either SGE1 or FTF1 mutants. WT: wild type; OE: over-expression transformants

**Fig. 5:**
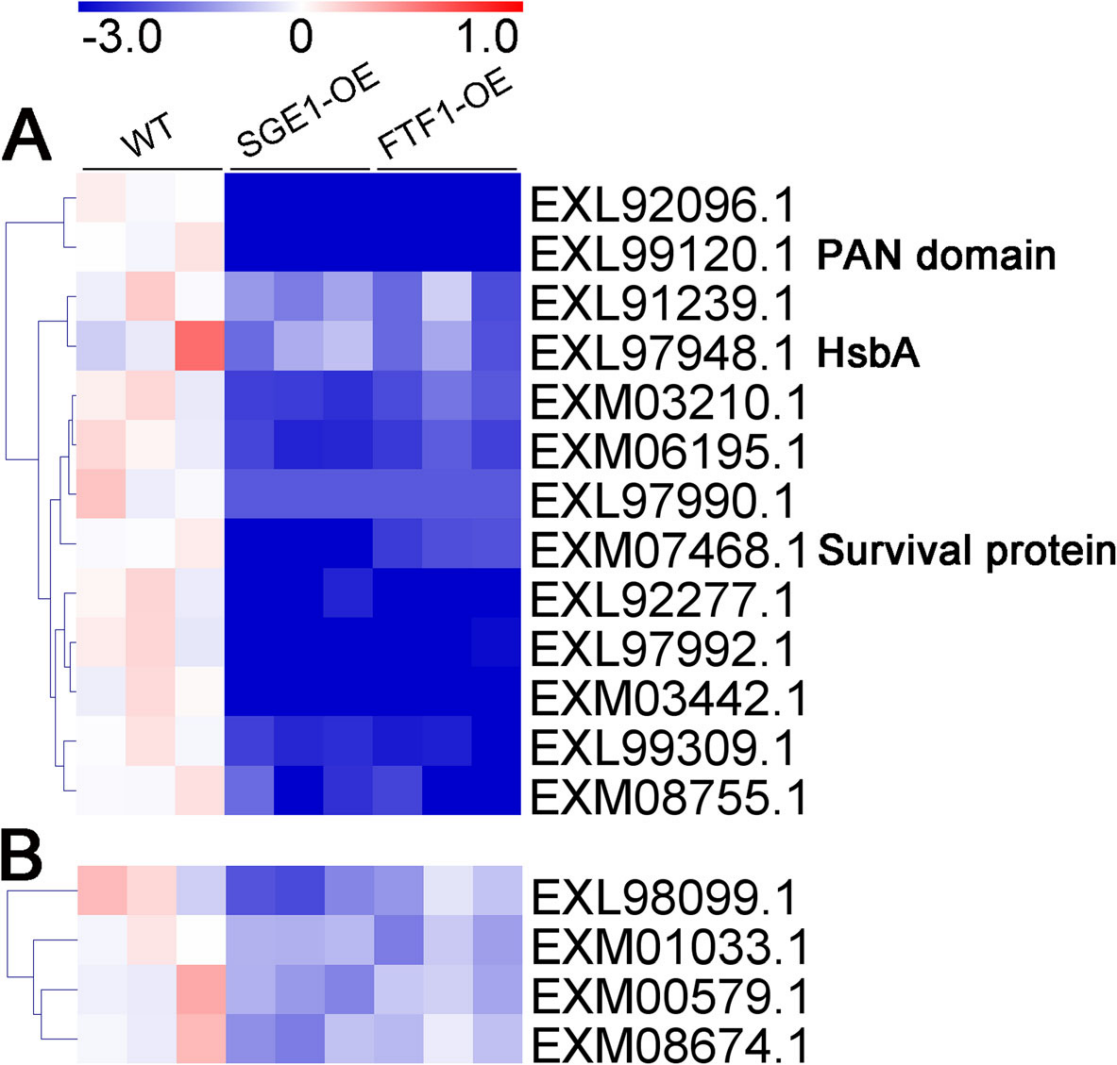
Profiles of the down-regulated effector candidates. Fold changes of protein abundance were calculated using the mean value of wild type samples as reference. The heatmaps were created based on the Log_2_(Fold change) values. **a** Proteins down-regulated in both over-expression samples. **b** Proteins down-regulated in either SGE1 or FTF1 mutants. WT: wild type; OE: over-expression transformants

**Fig. 6:**
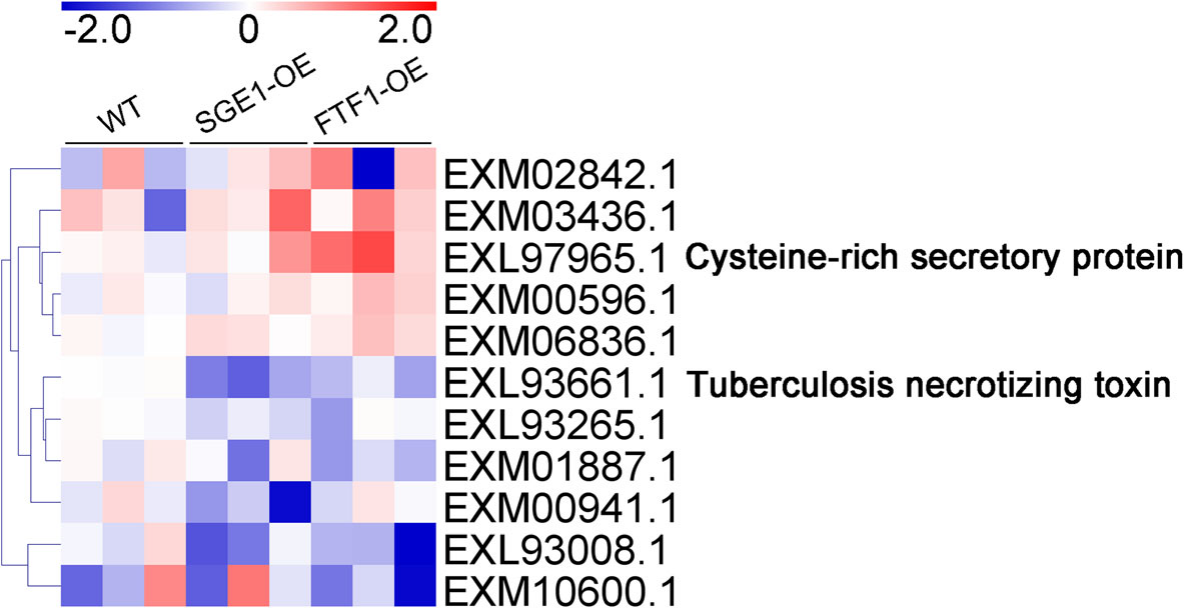
Profiles of the effector candidates with no significant change. Fold changes of protein abundance were calculated using the mean value of wild type samples as reference. The heatmaps were created based on the Log_2_(Fold change) values

**Fig. 7:**
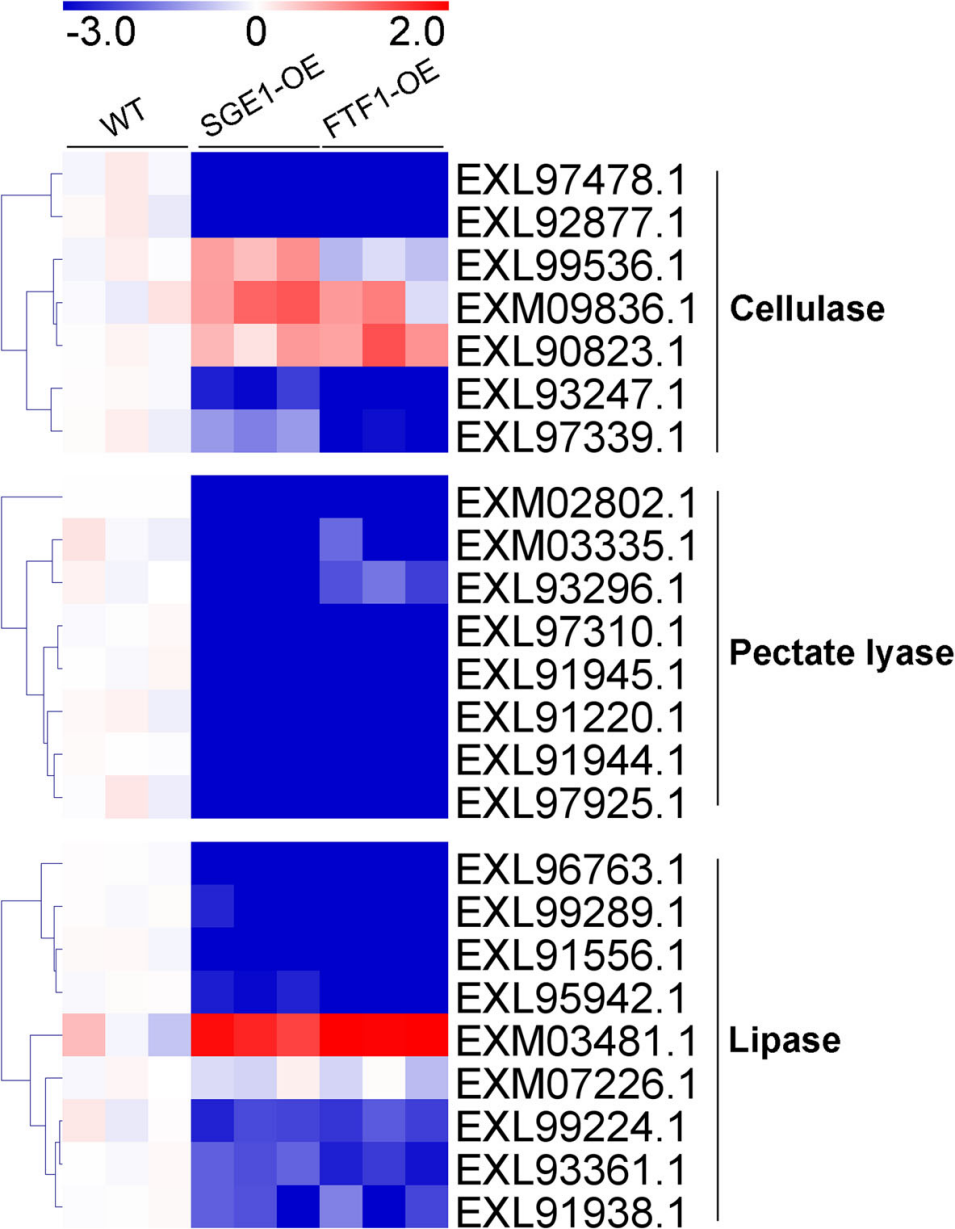
Profiles of host cell degrading enzymes. Fold changes of protein abundance were calculated using the mean value of wild type samples as reference. The heatmaps were created based on the Log_2_(Fold change) values

Differentially expressed proteins were defined as those that showed a fold change greater than 2.0 or less than 0.5 (|log_2_(Fold change)| > 1) based on the label-free quantitation. The 74 candidates were further classified into 4 clusters according to their abundance changes (Table S2). There were 29 proteins significantly up-regulated in both SGE1 and FTF1 OE samples (Fig. 4a), and 8 proteins up-regulated in either SGE1 OE or FTF1 OE samples compared with WT (Fig. 4b), including SIX6, SIX9, SIX13, a LysM effector, two Cerato-platanin effectors, and two Necrosis-inducing effectors. There were 13 proteins significantly down-regulated in both OE samples (Fig. 5a), and 4 proteins down-regulated in either SGE1 OE or FTF1 OE samples (Fig. 5b), including a PAM domain containing protein, a Hydrophobic surface binding protein A (HsbA), and a survival protein. Meanwhile, 11 proteins showed no difference among all three groups (Fig. 6). Besides, 9 proteins with extremely low abundance in all three groups were not taken into account for further analysis. In addition, there were 24 proteins identified as enzymes involved in host cell degrading; among these candidates, 19 proteins were significantly up-regulated and 1 protein down-regulated in both OE samples (Fig. 7).

### In silico promoter analysis

**Fig. 8:**
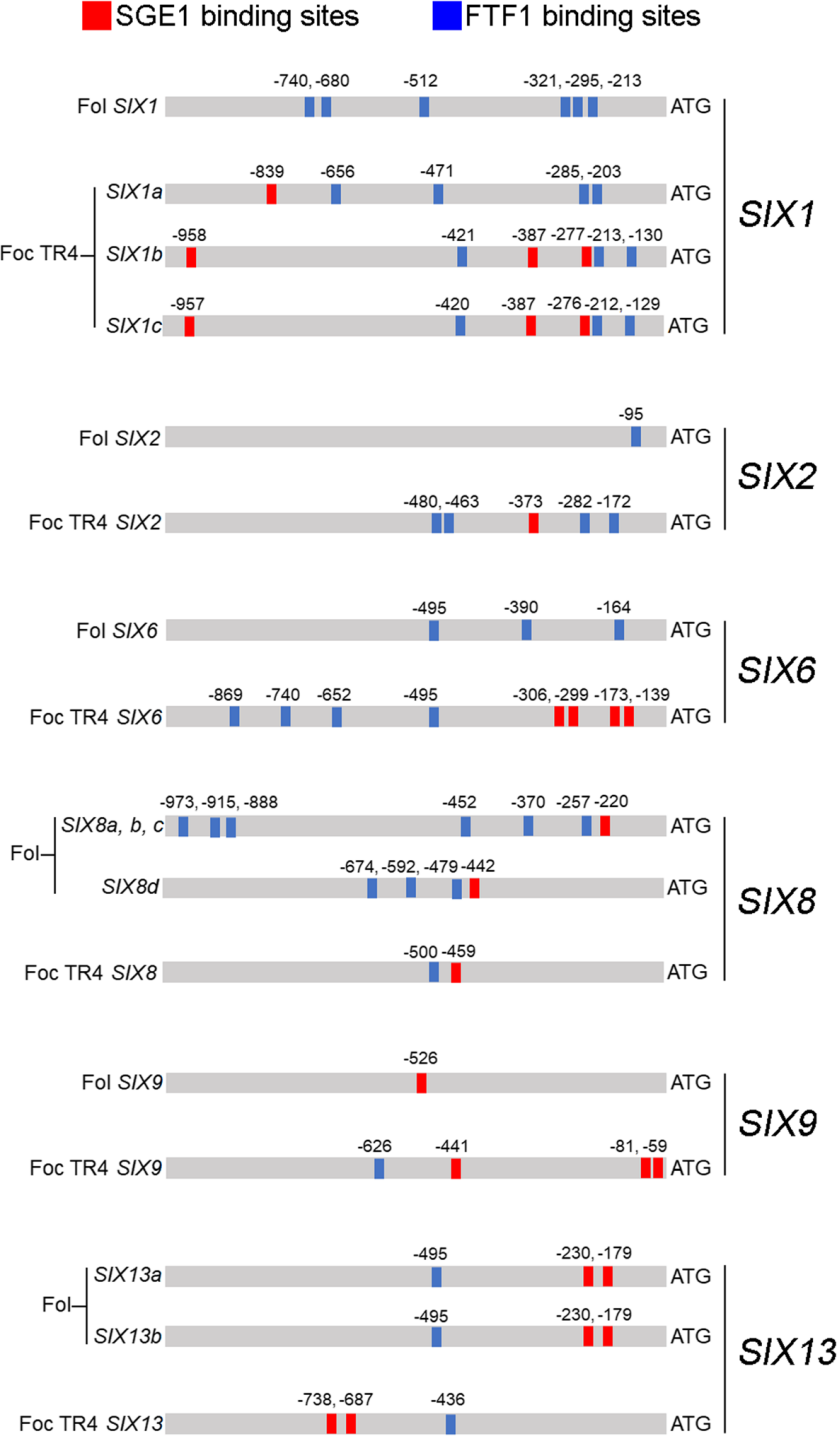
The promoter structures of *SIX* genes in *F. oxysporum* f. sp. *Cubense* tropical race 4 (Foc TR4) and *F. oxysporum* f. sp. *lycopersici* (Fol). Red boxes indicate SGE1 binding sites. Blue boxes indicate FTF1 binding sites. Single-letter code indicates the *SIX* gene homologues detected in each forma specialis

To find potential regulatory elements in the promoters of effector candidate genes, the 1000 bp upstream region of the genes were searched for the presence of 6mer TCGGCA, GGCAGT (FTF1 biding sites) and TAAAGT (SGE1 biding sites). The results showed that most of effector candidates contain at least one 6mer at the promoter regions, suggesting that these genes were directly regulated by SGE1 or/and FTF1 (Table S3). Investigation of the promoter regions of *SIX* orthologs of Foc TR4 and Fol showed that *SIX6* contains the most regulatory elements compared with other candidates, with 4 SGE1 binding sites and 4 FTF1 binding sites reside in the promoter region. Although SIX are highly conserved in *F. oxysporum* spp., there is variation in amount and location of regulatory elements between the orthologs of the two forma speciales (Fig. 8), suggesting that there is a different regulatory mechanism of effectors in Foc TR4 compared with Fol.

## Discussion

Identification of new effectors of plant pathogens become one of the research focuses in recent years. Unlike that in bacteria and oomycete, fungal effectors are usually diverse in protein features, making them difficult to be predicted and identified. Identification and functional analysis of effectors in Foc TR4, the destructive causal agent of banana wilt disease, are still inadequate till now. Most of fungal effectors showed in-planta expression patterns, such as the SIX effectors of *Fusarium* spp. [17, 19, 22]. Thus, successful simulation of in-planta status is a crucial step to induce the expression of effectors in vitro. According to the previous studies, transcription factors SGE1 and FTF1 function as key regulators in the expression of SIX [12, 13, 23], and the expression of these two transcription factors can also simulate an in-planta induction patterns [17]. So we reasoned that analysis of the secretome of the SGE1 and FTF1 constitutive expression strains could provide new clues for identification of new effectors. Then the SGE1 and FTF1 OE transformants were constructed respectively. After confirmation for the expression levels of *SGE1* and *FTF1* genes (Fig. 1), the transformants SGE1 OE3 and FTF1 OE1 were cultured with the modified Czapeck liquid to induce the expression of secreted proteins. Secreted proteins were precipitated from the culture supernatant with sodium deoxycholate and trichloric acetic acid. But the SDS-PAGE analysis showed that there were a lot of vertical streaking between protein bands (data not shown), indicating that the protein samples contained high portion of impurities. To remove the impurities, another purification step via Tris-HCl buffered phenol was conducted. After this step, protein samples with high purity were obtained (Fig. 2).

In our study, a total of 919 non-redundant proteins had been identified in the secretome of WT and OE strains (Table S1). But how to identify effectors from these 919 proteins was still a difficulty. Several criteria have been used for prediction of fungal effectors till now. Via comparative genomics incorporating the evolutionary signal of adaptation, 42 effector candidates have been identified in *Puccinia graminis f.sp. tritici* [24]. MITEs (Miniature Inverted-repeat Transposable Elements) in the promoter and downstream regions of effector genes could be used as criterion to identify new effector genes in *F. oxysporum* [25]. Using combined criteria, 78 effector candidates were identified in *Sclerotinia sclerotiorum* [26]. However, due to the diversity of fungal effectors, these models might just identify only a subset of the effector repertoire. Machine learning has recently been shown to be an efficient technique in effector prediction; and the program EffectorP was developed for prediction of fungal effectors [27, 28]. In the present study, the program EffectorP 2.0 was used to identify effector candidates from the secretome. The results showed that 180 of 919 proteins were predicted to be effector candidates. After that, the additional criteria were used to mine effectors from the 180 candidates: 1) proteins that contain secretion signals and are not apoplastic hydrolases were identified as effectors; 2) and proteins that do not contain secretion signals or conserved domains were also considered as effector candidates. After filtering with program SignalP 5.0 and Pfam database, 63 proteins with signal peptides and 11 proteins without signal peptides were identified as effector candidates of Foc TR4 (Fig. 3). Among these candidates, three SIX effectors, including SIX6, SIX9, SIX13, were identified; in addition, seven proteins were found to contain typical effector features, such as LysM, Cerato-platanin, Necrosis-inducing factor, and Cysteine-rich secretory protein (Table S2).

In the SGE1 and FTF1 OE transformants, 54 of the 74 candidates significantly changed in abundance. A total of 37 proteins were up-regulated in the OE samples (Fig. 4). In these candidates, the abundance of SIX6, SIX9, and SIX13 were up-regulated at least 2 fold; and 11 hypothetical proteins were significantly increased to over 8 fold in both two OE samples, including the typical LysM and Cerato-platanin effectors. The other 17 proteins were down-regulated in the OE samples (Fig. 5), with 7 candidates down-regulated over 8 fold in both OE samples. The results suggested that these genes are close related with the two transcription factors SGE1 and FTF1. Hemibiotrophic fungi possess a two-stage lifestyle: maintaining host viability at initial colonization and biotrophic growth; and eliciting host death for late necrotrophic growth. Genome-wide expression profiling revealed that virulence genes are transcribed in consecutive waves in *Colletotrichum* fungi: most effectors and secondary metabolism enzymes are induced at biotrophy stage, whereas most hydrolases and transporters are upregulated at necrotrophy stage [5, 29]. Thus we speculated that the 37 up-regulated effectors and 17 down-regulated effectors function at different stages of infection processes in Foc TR4. SGE1 and FTF1 play important roles in regulating the pathogenicity of *Fusarium* spp. [11, 13, 18, 19, 22, 30]. Knock-out of *SGE1* or silence of *FTF1* did not influence the penetration process of *Fusarium* species to their host, but impair the successful colonization to plant xylem [11, 19]. So it can be deduced that SGE1 and FTF1 play important roles in colonization of Foc TR4 to banana plant by up-regulating the biotrophy-associated genes and down-regulating the necrotrophy-associated genes. As mentioned above, in hemibiotrophic fungi, hydrolases are mainly expressed at the necrotrophic stage to destroy plant tissues. So the abundance of 24 identified enzymes involved in host cell structure degrading, including cellulase, pectate lyase, and lipase, were analyzed. As shown in Fig. 7, most of the enzymes were significantly down-regulated in both SGE1 OE and FTF1 OE strains, in accordance with our hypothesis that SGE1 and FTF1 facilitate colonization by promoting biotrophy and inhibiting necrotrophy of Foc TR4. However, one lipase (accession, EXM03481.1) was significantly up-regulated in the two OE groups, suggesting its special function in colonization process of Foc TR4.

The *Candida albicans* Wor1, the ortholog of the SGE1, could bind to the core motif [T (TAAAGT)TT] and regulate target genes [31]. FTF1 was predicted to bind to the element [TCGGCA] and [GGCAGT] [25]. Given these, the effector candidates were searched for the presence of conserved elements TAAAGT, TCGGCA and GGCAGT in the promoter regions. Our results showed that a total of 45 effector candidates contain at least one element in the promoters in the 37 up-regulated and 17 down-regulated effector candidates (Table S3). Moreover, SGE1 and FTF1 were all reported to regulate expression levels of *SIX* genes in Fol and *F. oxysporum* f. sp. *Phaseoli* (Fop) [11, 17–19]. But in the present study, SIX1, SIX2, SIX8 and SIX13 were not detected in secretome of the OE strains. Comparison of the promoter regions of *SIX* genes showed that the number and the locations of the elements are with a greater difference between Foc TR4 and Fol (Fig. 8). There are more elements presented in *SIX1*, *SIX2*, *SIX6* and *SIX9* promoters of Foc TR4 than that of Fol; and less elements in *SIX2*. For *SIX13*, although the number of the element are same, the locations are different (Fig. 8). In Fol and Fop, over-expression of SGE1 or FTF1 both significantly increase the transcription of *SIX1* [17, 19]; but in the present study, SIX1 were even not detected in the secretome of the OE strains. In addition, we found that SIX8 is required for the full pathogenicity of Foc TR4 to banana plant [22]; but SIX8 was not detected in the secretome either. These results suggested in addition to SGE1 and FTF1, there are other regulatory mechanisms for *SIX* gene expression in *F. oxysporum* spp.

## Conclusion

Taken together, through investigation of secretome via label free proteomics in combined with systematic analysis, a series of effector candidates under the regulation of SGE1 and FTF1 were identified in the present study. This work put a foundation for functional study of these newly identified effectors of Foc TR4.

## Methods

### Fungal strains and culture conditions

*F. oxysporum* f.sp. *cubense* race 4 (Foc TR4, isolate B2) strain, isolated in Hainan of China, was maintained on the PDA medium or Malt extract agar medium (Oxoid, Basingstoke, England) at 28 °C. For liquid culture, modified Czapeck liquid medium in which glucose was replaced with apple pectin were employed.

### Generation of over-expression strains

For generation of the over-expression strains of Foc TR4, the open reading frames (ORFs) of SGE1 and FTF1 (FOIG_16560; accession EXL90180.1) were cloned from cDNA. Briefly, RNA was isolated from the root of infected banana plantlets after inoculation for 7 days as described in our previous work [22]; then contaminating DNA was eliminated and cDNA was synthesized with Revert Aid First Strand cDNA Synthesis Kit (Thermo Fisher Scientific, MA, USA). Via fusion PCR, the ORFs without stop codon merged with the c-terminal 3XFALG coding sequence were amplified. The cloned ORFs-3XFLAG was checked by sequencing. To express the transcription factor genes in Foc TR4, the cloned ORFs-3XFLAG were ligated into the vector pFoNDHTN harboring promoter ToxA and hygromycin-resistant cassette. After that, Foc TR4 protoplasts were transformed with the linearized vectors and the correct transformants were identified as described in our previous work [22].

### Quantitative real-time PCR

RNA was extracted form Foc TR4 strains with CTAB method. The contaminating DNA was eliminated by using RNase-free DNase according to the manufacturer’s instruction (NEB, USA). After quantitation using NANODROP 2000 (Thermo Fisher Scientific, MA, USA), the first-strand cDNA was synthesized with Revert Aid First Strand cDNA Synthesis Kit (Thermo Fisher Scientific, MA, USA). The Quantitative real-time PCR was then performed using FastStart Universal SYBR Green Master (Roche, Switzerland) with a Light Cycler 96 Real-Time PCR System (Roche, Switzerland) instrument. The relative expression levels of the target genes were assessed based on 2^-ΔΔct^ method. *Actin* of Foc TR4 was employed as the internal control for gene expression analyses. Wild type was employed as the reference to calculate the expression level changes.

### Extraction and collection of secreted protein

To induce secretion of extracellular proteins, modified Czapeck liquid medium in which glucose was replaced with apple pectin was used to culture Foc TR4 strains. Conidia were collected from the fungal colonies cultured on PDA medium for 7 days, washed 2 times with ddH_2_O, and inoculated into 200 mL Czapeck liquid medium to make an initial concentration of 10^6^ conidia/mL. After incubation at 28 °C, 160 rpm for 3 days, the mycelium and conidia were removed from the culture by two runs of centrifugation, 6000 rpm for 10 min and 9000 rpm for 15 min respectively. Residual impurities was removed with 0.2 μm syringe filters. Then the proteins were precipitated from the supernatant: at first, sodium deoxycholate (Sigma-Aldrich, MO, USA) was added to the supernatant to the final concentration of 0.03% (w/v), followed by incubation on ice for 30 min; subsequently, trichloric acetic acid (100%) (Sigma-Aldrich, MO, USA) was added to the mixture to a final concentration of 10% (v/v), followed by incubation on ice for another 30 min. After that, the precipitated proteins were collected by centrifugation at 16,000 g at 4 °C for 30 min and washed 3 times with cold acetone. To remove residual apple pectin and other impurities in protein samples, the air-dried precipitate was re-dissolved in protein extraction buffer (0.5 M Tris-HCl, pH 8.3, 2% (v/v) NP-40, 20 mM MgCl_2_, 2% (v/v) β-mercaptoethanol, and 1 mM Phenylmethanesulfonyl fluoride); then an equal volume of Tris-HCl (pH 7.8) buffered phenol was added into the samples. The mixture was vortexed for 2 min, followed by incubation on ice for another 2 min; and the operation was repeated 5 times. After centrifugation at 16,000 g at 4 °C for 30 min, proteins were precipitated from the phenol phase with 5 vol of ice-cold saturated ammonium acetate in methanol overnight at − 20 °C. For the final step, the proteins were collected by centrifugation at 16,000 g at 4 °C for 30 min, washed with cold saturated ammonium acetate in methanol and acetone, and air-dried.

### Peptide preparation for mass spectrometry

In the present study, a total of three biological replicates for each of the groups were used for proteomics analysis. Air-dried protein samples were re-dissolved in buffer containing 0.1 M triethylammonium bicarbonate (TEAB, pH 8.5) and 6 M urea. Protein concentration was determined by Bradford protein assay. Then supernatant from each sample, containing precisely 0.12 mg of protein was digested with Trypsin Gold (Promega) at 1:50 enzyme-to-substrate ratio. After 16 h of digestion at 37 °C, peptides were desalted with C18 cartridge to remove the high urea, and desalted peptides were dried by vacuum centrifugation.

### LC-MS/MS analysis

The LC-MS/MS Analysis was conducted by Novogene (Beijing, China). The EASY-nLCTM 1200 UHPLC system (Thermo Fisher) was applied for separation of the tryptic peptide mixtures. A sample volume containing 2 μg of total peptides was injected onto a home-made C18 Nano-Trap column (2 cm × 100 μm, 3 μm) and separated on a home-made analytical column (15 cm × 150 μm, 1.9 μm), using a 60 min linear gradient from 5 to 100% eluent B (0.1% FA in 80% ACN) in eluent A (0.1% FA in H_2_O) at a flow rate of 600 nL/min. After separation, the tryptic peptide mixture was analyzed simultaneously with a Q-Exactive HF-X mass spectrometer. The MS was operated in positive polarity mode with a scan range of 350 to 1500 m/z. Full MS scans range from 350 to 1500 m/z were acquired at a resolution of 60,000 (at 200 m/z) with an automatic gain control (AGC) target value of 3 × 10^6^ and a maximum ion injection time of 20 ms. Then the 40 most abundant precursor ions from full MS scan were selected for fragmentation using higher energy collisional dissociation (HCD) fragment analysis at a resolution of 15,000 (at 200 m/z) with an AGC target value of 1 × 10^5^, a maximum ion injection time of 45 ms, a normalized collision energy of 28%, an intensity threshold of 2.2e4, and the dynamic exclusion parameter of 20 s.

### Protein identification and relative quantitation

The resulting spectra from each fraction were searched separately against Foc TR4 protein database (NCBI: txid1089451) by the search engines Proteome Discoverer 2.2 (PD 2.2, thermo). The searched parameters as follows: 10 ppm for precursor ion scans, 0.02 Da for the product ion scans, Carbamidomethyl as fixed modifications, Oxidation of methionine (M) and acetylation of the N-terminus as variable modifications, 2 for mis-cleavage sites. For protein identification, protein with at least 1 unique peptide was identified at FDR less than 1.0% on peptide and protein level, respectively. Proteins containing similar peptides and could not be distinguished based on MS/MS analysis were grouped separately as protein groups. Precursor quantitation based on intensity was used for label-free quantitation. The 90th and 95th percentiles of abundance of all the detected proteins were 423, 295 and 646, 006, respectively; so the values of ‘protein abundance + 500, 000’ were used for calculating the relative Fold change and Log_2_(Fold change) of the candidates. The protein quantitation results were statistically analyzed by one way ANOVA test, the significant difference defined as fold change greater than 2.0 or less than 0.5 (|log_2_(Fold change)| > 1) was used to screen the differentially expressed proteins**.** The proteomics data were deposited in the iProx database: IPX0001783000.

### The functional analysis of protein, prediction of effectors, and prediction of transcription factor binding sites

Effectors were predicted using the EffectorP 2.0 sever (http://effectorp.csiro.au/) [28]. Signal peptides were predicted with SignalP 5.0 [32]. Protein function analysis was conducted against the Pfam protein database. The 1000 bp upstream region of the predicted effector genes were searching for the presence of regulatory elements TCGGCA, GGCAGT (as the FTF1 biding sites) and TAAAGT (as the SGE1 biding sites).

## Supplementary information

**Additional file 1 Table S1.** Proteins detected in the secretome through LC-MS/MS analysis.

**Additional file 2 Table S2.** Classification of effector candidates based on their abundance changes.

**Additional file 3 Table S3.** Predicted binding sites of SGE1 and FTF1 in the promoters of effector candidates.

## Data Availability

The datasets generated during the current study are available in the iProx database IPX0001783000. The materials used during the current study are available from the corresponding author on reasonable request.

## Abbreviations

Fol: *F. oxysporum* f. sp. *Lycopersici*
Foc: *F. oxysporum* f. sp. *Cubense*
Fop: *F. oxysporum* f. sp. *Phaseoli*
SIX: Secreted in xylem
SGE1: SIX gene expression 1
FTF: Fusarium transcription factor
OE: Over-expression
HCD: Higher energy collisional dissociation
